# Age-specific distribution of intraocular pressure in elderly Iranian population and its associated factors

**DOI:** 10.22088/cjim.14.1.112

**Published:** 2023

**Authors:** Mohammad Javad Ghanbarnia, Nour Mohammad Panahi, Seyed Ahmad Rasoulinejad, Seyed Reza Hosseini, Hoda Shirafkan, Gholam Abbas Roustaei, Ebrahim Mekaniki, Mehrnoosh Ghasemi, Reza Ghadimi, Ali Bijani

**Affiliations:** 1 Student Research Committee, Health Research Center, Babol University of Medical Sciences, Babol, Iran; 2 Department of Ophthalmology, Ayatollah Rouhani Hospital, Babol University of Medical Sciences, Babol, Iran; 3 Social Determinants of Health Research Center, Health Research Institute, Babol University of Medical Sciences, Babol, Iran

**Keywords:** Intraocular pressure, Intraocular hypertension, Ocular tonometry, Glaucoma, Cohort study, Body mass index, Diabetes mellitus, Blood pressure

## Abstract

**Background:**

The purpose of this study was to determine the distribution of intraocular pressure (IOP) and assess its association with age, sex, systemic blood pressure, diabetes mellitus, body mass index (BMI) and tobacco smoking in Iranian elderly population.

**Methods:**

This cohort-based, cross-sectional study assessed elderly individuals aged 60-90 years in Amirkola, northern Iran, in 2016-2017. Past medical history, blood pressure, diabetes mellitus, BMI and tobacco smoking were recorded through an interview and physical examination. IOP was assessed using non-contact tonometry.

**Results:**

Total of 1377 individuals participated in this study, out of which 1346 IOP measurements were included for the final analysis. The mean age of participants was 69.4 ± 7.1 years and mean IOP was determined to be 16.7 ± 3.2 mmHg. Majority of the participants were males (56.1% vs 43.1%), 73.8% of participants were overweight or obese, 6.1% smoked tobacco, 28.9% had diabetes mellitus and 84.9% had higher than normal blood pressure. Through multiple regression analysis, it was determined that age (β=-0.132, p<0.001) was negatively associated with IOP, and the presence of diabetes mellitus (β=0.118, p<0.001), systolic blood pressure (β=0.101, p<0.001), and BMI (β=0.020, P=0.020) were positively associated with IOP.

**Conclusion:**

Mean IOP of individuals in this study was higher than average based on other studies. Age, was negatively and systemic blood pressure, BMI and presence of diabetes mellitus were positively associated with mean IOP of elderly Iranian population. Sex and tobacco smoking were not correlated with IOP.

Normal intraocular pressure (IOP) is an important property of the eye through which structural support is provided and physiological and pathophysiological processes are maintained (1,2). The balanced equilibrium between aqueous humor secretion by the ciliary body and its drainage through trabecular meshwork and uveoscleral pathway corresponds to a normal IOP (3). Aqueous humor equilibrium deviations, hence has detrimental effects on the eye. Increased IOP is a major risk factor for the development and progression of glaucoma (4, 5). Glaucoma is estimated to have affected 76 million people in 2020 worldwide and it is projected to effect 112 million people by 2040, making it the main culprit of irreversible vision loss in the world (6-8). Because of its significant worldwide prevalence and irreversible nature, glaucoma has been a subject of various studies that have indicated its risk factors such as ageing, sex, ethnic background, family history, genetic disposition, anatomical characteristics and increased IOP (3, 9-11). 

However, because of its modifiability, IOP pressure still remains as the only proven factor that can halt or suppress progression of glaucoma (11-13). Therefore, IOP is still used as an essential screening tool for prevention, early diagnosis and treatment follow-up target.

Investigating normal variations of IOP among populations has been a subject of various studies for decades (14,15). These studies have clearly shown that a definite normal IOP value range cannot be applied to individuals universally, because normal IOP distributions in different geographical populations are varied with respect to age, sex, ethnicity and systemic factors including blood pressure, body mass index and accompanying medical conditions (16-18). Therefore, several population-based studies have explored distribution of IOP in various geographical locations enabling health practitioners to set individualized target IOP for patients. Most of these studies were carried out in American, European, African and East and South Asian populations of (16-21). However, relevant population-based study in Iran has been limited. Prior two studies concluded that mean IOP of Iranians is lower than the worldwide average and listed diabetes, high blood pressure and obesity as important risk factors (22-23). However, target population of both of these studies focused mainly on individuals younger than 65 years old, hence highlighting the lack of evidence from elderly Iranian population. As a result, we explored the distribution of IOP among the individuals from an elderly population of Northern Iran with respect to ageing, diabetes mellitus, systemic blood pressure, tobacco smoking and BMI. 

## Methods

This cross-sectional study was conducted as part of the second phase of the Amirkola Health and Ageing Project (AHAP) (24). The AHAP is a comprehensive cohort study of Northern Iranian elderly population. Its second phase took place between 2016 and 2017, during which, thorough eye examinations for the current study were carried out (24). Participants in this study were all older than 60 years of age and had resided in Amirkola, in Northern Iran. Individuals who had been previously diagnosed with glaucoma or ocular hypertension used IOP-lowering ophthalmic drops, individuals with previous ocular surgery, and those with incomplete examination and data were excluded from the study. Furthermore, individuals identified as suspects for having glaucoma or glaucomatous optic nerve after slit lamp examination, were excluded. The Research Ethics Committee of Babol University of Medical Sciences approved this study (IR.MUBABOL.HRI.REC.1400.142). All aspects of the study were in accordance with the tenets of the Helsinki Declaration. 

Upon signing an informed consent form, participants underwent physical and ophthalmologic examination. A questionnaire was utilized to obtain demographics data including age, sex, medications taken including ophthalmic drops, history of past medical conditions, past eye surgeries and tobacco smoking. Current tobacco smokers were placed in smoking category. Thorough physical and ophthalmologic examinations were carried out by trained health practitioners and ophthalmologists. Identification of diabetes mellitus were based on previous diagnosis by a specialist or fasting blood sugar higher than 126 mg/dL measured on two occasions. Body mass index (BMI) was calculated by dividing weight(kilograms) by height (meters square). Participants were then placed in subcategories such as underweight (BMI<18.5kg/m^2^), normal (18.5≤BMI≤24.9kg/m^2^), overweight (25.0≤BMI≤29.9kg/m^2^), obese (BMI≥30 kg/m^2^). Omron M7 blood pressure monitor was utilized to record participants’ blood pressure in a relaxed sitting position. Blood pressure values were defined as normal (sBP<120 mmHg or dBP<80 mmHg), elevated (120≤sBP≤129 mmHg or dBP<80 mmHg), hypertension stage 1 (130≤sBP≤139 mmHg or 80≤dBP≤89 mmHg) and hypertension stage 2 (sBP>140 mmHg or dBP> 90 mmHg). Slit lamp examination was followed by pupil dilation using Tropicamide 1% and retina and optic disk were assessed. TOPCON non-contact tonometry was used to record IOP. Both eyes were examined and IOP was measured three times and the average out of three measurements was recorded for each eye. Higher IOP out of the two eyes was then presented in results. 

Statistical analysis was performed on SPSS software (Statistical Package for Social Science, Version 25.0; IBM-SPSS Inc, Chicago, Illinois, USA). Independent sample t-test was used to determine significant differences in mean IOP of binary variables (sex, DM, smoking). To determine significant difference between mean IOP of different subgroups (subgroups of age, BMI, blood pressure) ANOVA was used. Two-tailed p-values were considered significant if <0.05. To further investigate the effect of variables (increase or decrease) on mean IOP in a multivariate model, first, univariate linear regression and then backward multivariate linear regression were utilized. IOP was set as dependent variable in regression models. Unstandardized coefficient(B) and standardized coefficient(β) were determined and p-value less than 0.05 was set to be significant. IOP distribution percentiles stratified by age and sex was then presented. 

## Results

Phase two of Amirkola Health and Ageing Project (AHAP) included 1377 elderly participants. After applying the exclusion criteria, 1346 individuals entered the study. Table 1 shows the characteristics of the study participants. Mean IOP was 16.7±3.2 mmHg (range 7-35 mmHg). The mean age of participants was 69.4±7.1 years (range 60-95 years). 58.5% of the participants were from 60 to 70 years old and the highest mean IOP belonged to the 60-65 age category (17.1±3.2 mmHg). Difference between mean IOP of different age subgroups was significant (p<0.001). 

This study had a higher male population compared to female (56.1% vs 43.1%) and mean IOP was slightly higher in female participants (16.8±3.1 mmHg vs 16.6±3.2 mmHg) but not significant (P=0.131). The mean BMI of the participants was 28.2±4.8 and the highest IOP belonged to underweight category (18.1±3.7 mmHg), however only a small number of the participants (1.1% of participants) were placed in this category. Significant difference between mean IOP of different BMI subgroups was observed (p<0.001). Significant difference between IOP of tobacco smokers and non-smokers was not found (P=0.908). Almost one third (28.9%) of the participants had diabetes mellitus and mean IOP was significantly higher in diabetic individuals (17.4±3.2 mmHg vs 16.4±3.1 mmHg, p<0.001). Mean systolic blood pressure was found to be 141.5±21.3 mmHg and mean diastolic blood pressure was 83.4±12.1 mmHg. In sub-groups of both systolic and diastolic blood pressure, the highest IOP belonged to individuals with stage 2 hypertension (16.8±3.3 mmHg in systolic category and 17.0 ±3.1 mmHg in diastolic category). 

Age, sex, tobacco smoking, BMI, diabetes mellitus, systolic and diastolic blood pressure were analyzed in univariate and multivariate linear regression as shown in table 2. IOP was set as dependent variable in this analysis. Sex and tobacco smoking were not significantly associated with IOP (P=0.131 and P=8.898), hence they were not included in multiple regression model. Diastolic pressure was significantly associated with IOP (P=0.006), however because it paralleled the effects of systolic blood pressure, it was removed in backward multivariate regression. Age was negatively associated with IOP (standardized coefficient(β)= -0.0132) and this association was significant (p<0.001). Presence of diabetes mellitus, systolic blood pressure and BMI were positively associated with IOP (β= 0.118 p<0.001, β= 0.101 p<0.001, β=0.065 P=0.020). Distribution of IOP stratified by sex and age group is demonstrated in table 3. IOP 95th percentile for both male and female groups of all ages were identical (22 mmHg). IOP 95th percentile for the age group 60-64 was the highest (24 mmHg). 

**Table.1:** Mean IOP with respect to participants demographics and characteristics

**Variables**	**No. of Participants (%)**	**Mean IOP mmHg ± SD**	**P-value**
IOP			
Mean ± SD (Range)	16.7 ± 3.2 (7-35)		
Age			
Mean ± SD(Range)	69.4 ± 7.1(60-95)		
Age Category			<0.001
60-64	368(26.7%)	17.1 ± 3.2	
65-69	438(31.8%)	16.9 ± 3.0	
70-74	256(18.6%)	16.6 ± 3.0	
75-79	170(12.3%)	16.1 ± 3.5	
80-84	101(7.3%)	15.8 ± 3.3	
+85	44(3.2%)	15.7 ± 3.4	
Sex			0.131
Male	772(56.1%)	16.6 ± 3.2	
Female	605(43.1%)	16.8 ± 3.1	
BMI			
Mean ± SD (Range)	28.2 ± 4.8 (15.3-44.7)		
BMI Category			<0.001
Underweight	15(1.1%)	18.1 ± 3.7	
Normal	345(25.1%)	16.1 ± 3.2	
Overweight	567(41.2%)	16.6 ± 3.3	
Obese	449(32.6%)	17.2 ± 2.9	
Smoking			0.908
Yes	84(6.1%)	16.7 ± 3.5	
No	1293(93.9)	16.7 ±3.2	
Diabetes Mellitus			<0.001
Yes	398(28.9%)	17.4 ± 3.2	
No	979(71.1%)	16.4 ± 3.1	
Systolic Blood Pressure (mmHg)			
Mean ± SD(Range)	141.5 ± 21.3(78-215)		
Systolic Blood Pressure Categories			0.110
Normal	187(13.4%)	16.2 ± 3.2	
Elevated	236(16.9%)	16.6 ± 2.9	
HTN. Stage 1	264(18.9%)	16.6 ± 3.0	
HTN. Stage 2	687(49.1%)	16.8 ± 3.3	
Diastolic Blood pressure			
Mean ± SD(Range)	83.4 ± 12.1(40-135)		
Diastolic Blood Pressure Categories			0.027
Normal and Elevated	537(38.4%)	16.4 ± 3.3	
HTN. stage1	465(33.2%)	16.7 ± 3.1	
HTN. Stage 2	372(26.6%)	17.0 ± 3.1	

IOP= Intraocular Pressure SD= Standard Deviation BMI= Body Mass Index

**Table. 2:** Relationship of age, sex, Tobacco smoking, Diabetes Mellitus, systolic and diastolic blood pressure with IOP analyzed by Univariate and Multivariate Linear regression

		**Univariable Linear Regression**			**Multivariant Linear Regression**		
		**B**	**β**	**p-Value**	**B**	**β**	**P-value**
Age		-0.061	-0.135	<0.001	-0.059	-0.132	<0.001
Sex							
Male		Reference	Reference	Reference			
Female		0.264	0.041	0.131			
Smoking							
Yes		0.047	0.004	8.898			
No		Reference	Reference	Reference			
Diabetes Mellitus							
Yes		0.986	0.140	<0.001	0.829	0.118	<0.001
No		Reference	Reference	Reference	Reference	Reference	Reference
Systolic Blood Pressure		0.014	0.091	0.001	0.015	0.101	<0.001
Diastolic Blood Pressure		0.020	0.075	0.006			
BMI		0.077	0.116	0.000	0.043	0.065	0.020

B= Unstandardized Coefficient β= Standardized Coefficient

**Table 3:** IOP Distribution percentiles stratified by age and sex

			**Percentiles**					
**Variables**	**Mean**	**SD**	**5** ^th^	**25** ^th^	**50** ^th^	**75** ^th^	**95** ^th^	**99** ^th^
Sex								
Male	16.57	3.24	12	14	16	18	22	28
Female	16.84	3.10	12	15	17	18	22	27
Age Category								
60-64	17.14	3.15	13	15	17	19	24	27
65-69	16.89	3.04	12	15	17	18	21	27
70-74	16.59	3.03	12	14	17	19	22	25
75-79	16.10	3.47	11	14	16	18	22	27
80-84	15.80	3.31	11	14	16	17	21	28
+85	15.66	3.31	10	14	16	19	21	21

## Discussion

This cohort-based cross-sectional study is the first study that explored population based IOP in an elderly population comprising only individuals older than 60 years of age. Our reported mean IOP was significantly lower than the three previous studies conducted on Iranian population including Tehran eye study, Shahroud eye study and Yazd eye study (16.7 ±3.2 mmHg vs 14.5 ±2.6 mmHg, 12.9 ±2.3 mmHg and 14.2±2.5 respectively) (22, 23, 25). However, in this study, the mean age of participating population was significantly higher compared to the previous studies (69.4±7.1 years vs 33.6±17.3 years, 50.9±6.2 years and 53.1±9.6 years respectively). 

Because of the insufficient data evidence of elderly individuals in previous studies, precise comparison of the mean IOP of this study with respect to Iranian population cannot be properly derived. It is, however, still plausible to assume justification of higher mean IOP in this study by correlating it to advanced age and higher rate of chronic systemic disorders including higher blood pressure and diabetes mellitus. Comparing our results with those obtained by worldwide studies which had similar age characteristics, revealed that our mean IOP was still higher than most studies. For instance, Blue Mountain eye study in Australia, Epic Norfolk eye study in Britain, Thessaloniki eye study in Greece and Beijing eye study in China all had mean population health of over 60 years and they all reported lower IOP than our study (16.0 mmHg, 16.0 mmHg, 15.0 mmHg and 14.7 mmHg respectively) (26-29). The mean IOP in this study was lower compared to results obtained from Barbados eye study and Baltimore eye survey in the USA (18.7mmHg and 17.2mmHg) (30, 31). Lack of studies that solely focused on elderly population and also inconsistency of the few available studies, make it difficult to establish definite logical connection between mean IOP of our study population to the rest. However, inverted U-shaped trend in IOP change, with respect to age that was suggested in a meta-analysis of IOP in European population-based studies is a plausible explanation to the higher IOP in this study (32). 

**Fig. 1:**
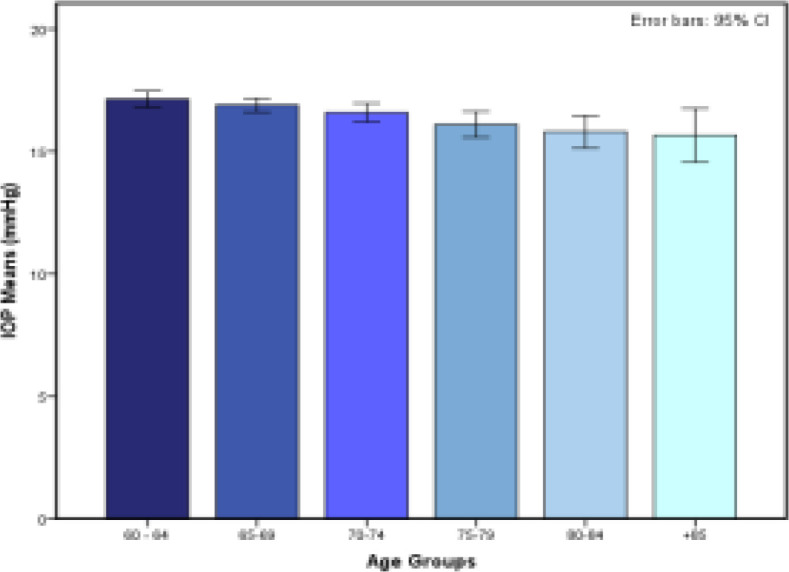
Distribution of mean IOP in different age groups

According to this inverted U shape pattern, IOP increases until the seventh decade of life, reaching a plateau, and decreases after 70 years of age. 58.5% of participants in our studies are 60-70 years old which have the highest mean IOP among age groups, hence, increasing the overall mean IOP of this study. Further investigation of this study cohort in future follow-ups and studies from various parts of Iran is required to help solve the dilemma that whether mean IOP of northern Iranian population is higher than the rest of Iranian population or mean IOP of Iranian elderly in general is higher than various parts of the world. Comparing the trends of changes in populations’ mean IOP with respect to age, in contrast to comparing the overall mean IOP of populations, gives a more accurate representation of the differences in IOP in various populations. Furthermore, this helps establish a universal pattern of IOP changes in response to advancing age. In our study, age and IOP had an inverse relationship which resulted in IOP decreasing steadily as we moved up to the age groups. The rate at which IOP decreased, decelerated after 85 years of age as shown in figure 1. Multivariate linear regression revealed that for every decade increase in age, IOP dropped by 0.6 mmHg as shown in table. 2. Our finding is consistent with several other studies including Beijing eye study that reported a 0.5 mmHg drop in IOP with every decade increase of age and further investigations in Chinese, South Korean, Japanese populations also reported inverse relationship of age and IOP (29, 33-35). 

This is in contrast to all three previous Iranian population-based studies that demonstrated a positive correlation between IOP and age (22 ,23, 25). However, all three of these studies comprised populations of significantly lower mean age compared to the current study. In the light of recent studies, the pattern of inverse U shape in demonstrating the association between age and IOP has become more prominent as it has been able to rationalize the ever-lasting discrepancies and inconsistencies in reports of age and IOP relationship in various study populations (27, 32).

 Individuals younger than 60 years of age were not included in our study. However, assuming our current elderly population study results to be consistent and continuous with the three aforementioned younger-population Iranian studies, it is highly likely that our data depicted the descending arm of the inverse U-shape pattern where IOP decreased steadily with advancing age after reaching a plateau in the seventh decade of life. 

**Fig. 2:**
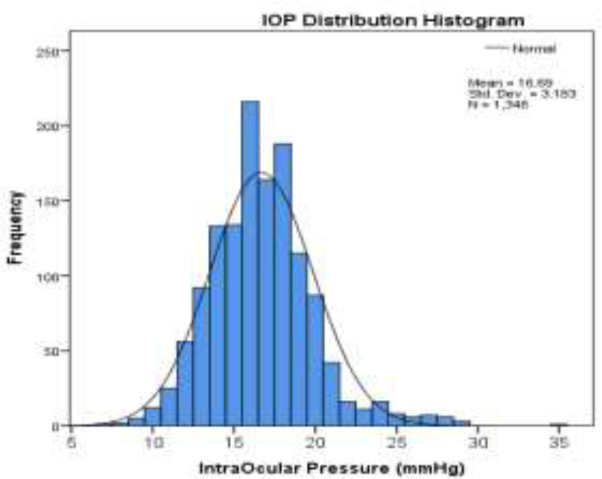
Distribution of IOP in elderly population of Amirkola Health and Ageing Project(AHAP)

Our study indicated a mostly normal Gaussian-like distribution of IOP, as illustrated in figure 2, which is consistent with previous studies (22, 23, 25). In this study, 5.5% of the participants were noted to have IOP above 21 mmHg indicating these individuals as suspects of intraocular hypertension that require further investigation. However, given the fact that the mean IOP of this study was higher than most studies, designating the same traditional cut off limit of 21 mmHg as upper normal limit of IOP seems inappropriate. Therefore, sex and age specific distribution of IOP and their respective percentiles as shown in table 3 should be taken into account when considering suspects for intraocular hypertension and glaucoma in future studies. 

The literature has been inconsistent in establishing correlation between IOP and sex. In our study, significant association between sex and IOP was not observed (P= 0.131 in univariate regression). These results concur with several studies including Yazd and Tehran eye studies on Iranian population, Saudi Arabia study and Japanese studies (22, 25,36, 37). On the other hand, higher IOP in women compared to men were observed in various studies (23, 30, 33, 38). Sex differences in IOP has been attributed to menopause hormonal changes in women and its subsequent effect on IOP-lowering agents including estrogen and nitric oxide (39). Similar to sex, tobacco smoking was not associated with IOP in univariate regression and was not included in multivariate linear regression (P=8.898). Similar to our results, several studies identified no association between IOP and tobacco smoking (21, 27, 40). However, some studies reported positive association between tobacco smoking and IOP (25, 29). A probable explanation for this discrepancy is the method used to define tobacco smoking which is different in every study. Our study included individuals who, at the time of this study, smoked more than 7 cigarettes per week in the tobacco smoking category and did not account for those who quit smoking. Significant positive association between systemic blood pressure and IOP was observed in this study. Systolic blood pressure was significantly associated with IOP in linear regression analysis (p-value 0.001) and this significance was also observed in multivariate regression model. Our results indicate that for every 10 mmHg increase in systolic blood pressure, IOP increases by 0.15 mmHg. Evidence of positive association between blood pressure and IOP is well established in the literature including studies from American, Chinese, Japanese, Barbadian, Indian, Iranian, British and Russian populations (1, 16-17, 21, 23, 27, 29, 34, 60). Increase in IOP has been attributed to the ciliary artery pressure increase as a result of elevated blood pressure which leads to increased ultrafiltration and overproduction of aqueous humor. These findings hinted at propositions that lowering systemic blood pressure could potentially lower glaucoma risk through reduction of IOP (19, 41). On the other hand, various studies indicated the role of reduced ocular perfusion pressure as a risk factor of open angle glaucoma and glaucomatous optic neuropathy (27, 42). These findings highlight the complex nature of glaucoma and its multifactorial associations. Even though the positive association between systemic blood pressure and IOP has been established, determining positive association between systemic blood pressure and glaucoma requires further investigation.

Similar to SBP, the literature has consistently presented BMI as another major risk factor for developing high IOP (1, 16-19, 21, 25, 32, 38, 40, 41). Our study also further validated these findings as it indicated positive association between BMI and IOP. Both univariate and multivariate regression analysis demonstrated a significant association (p<0.001and P= 0.020). Despite the fact that higher BMIs were found to be associated with higher IOP, the underweight BMI group (BMI<18.5) presented the highest mean IOP (18.1±3.7 mmHg). However, only 1.1%(n=15) of the participants were categorized as underweight, making their contribution to our study population overall BMI and IOP insignificant. Our results indicate that for every one unit increase in BMI, IOP increases by 0.04 mmHg. Some studies related this trend to a higher degree of induced Valsalva maneuver during eye examination in individuals with higher BMI (32).

 However, this hypothesis is not reconcilable; given the fact that positive BMI and IOP trend exists both in lower and higher BMI categories. Moreover, our IOP examination is done using an NCT as opposed to Goldman applanation using a slit lamp which further reduces probability of this hypothesis. Even though the definite mechanism of this trend is still unclear, a more plausible explanation is elevated episcleral venous pressure and reduced outflow as a result of increased in periorbital and intraorbital fat tissue in individuals with higher BMI (30, 32, 41). Our study demonstrated a positive association between diabetes mellitus and IOP. Several studies have indicated diabetes mellitus as a risk factor for developing glaucoma (43, 44). A meta-analysis concluded that every year that passes from diagnosis, diabetes mellitus increases the risk of developing glaucoma by 5% (43). However, the literature results are inconsistent when it comes to association between IOP and diabetes mellitus. Some studies reported no significant association (21, 25). 

While, consistent with our results, most studies concluded a positive association between IOP and the presence of diabetes mellitus (16, 18, 19, 23, 40, 43). IOP increase in diabetic individuals has been attributed to osmotic gradient caused by hyperglycemia which pushes aqueous humor into the anterior chamber (43, 44). Moreover, through vascular damage, diabetes mellitus can cause various ocular diseases including glaucoma independent of IOP increase (43). 

Potential limitations of this study should be addressed. First of all, this study did not include individuals younger than 60 years of age. Despite the fact that younger individuals were not the main focus of this study, including younger participants could have provided a better understanding of the trends in IOP changes with respect to age in northern Iranian population as a whole. Utilizing non-contact tonometry as opposed to Goldman applanation is another potential limitation of this study. Even though both techniques have been used in various studies, previous Iranian studies utilized Goldman applanation tonometry (22, 24). Cross-sectional nature of this study as opposed to a longitudinal design poses another potential limitation. However, given that this study is part of a cohort project, future research can be conducted implementing a longitudinal approach. 

This project was a comprehensive ophthalmology study that focused solely on elderly Iranian population and their eye health. We aimed to bridge the gap between studies that previously involved younger population and underrepresented elderly populations. Our study demonstrated that mean IOP in elderly Iranian population is higher than reported average in other studies.

 It also indicated a positive association between IOP and systemic blood pressure, diabetes mellitus, BMI and a negative association with age. Our findings in this study not only help better understand IOP changes in older age groups but they are also significant for improving public health through the prevention and the early detection of glaucoma. Future investigations can implement a more comprehensive ophthalmologic examination and also a longitudinal study design as opposed to cross-sectional. 
